# Repurposing Pilocarpine Hydrochloride for Treatment of Candida albicans Infections

**DOI:** 10.1128/mSphere.00689-18

**Published:** 2019-01-23

**Authors:** Christopher Nile, Monica Falleni, Daniela Cirasola, Abeer Alghamdi, Oliver F. Anderson, Christopher Delaney, Gordon Ramage, Emerenziana Ottaviano, Delfina Tosi, Gaetano Bulfamante, Giulia Morace, Elisa Borghi

**Affiliations:** a Oral Sciences Research Group, University of Glasgow Dental School, School of Medicine, Dentistry and Nursing, College of Medical, Veterinary and Life Sciences, University of Glasgow, Glasgow, United Kingdom; b Division of Human Pathology, Department of Health Sciences, Università degli Studi di Milano, Milan, Italy; c Laboratory of Microbiology, Department of Health Sciences, Università degli Studi di Milano, Milan, Italy; Duke University Medical Center

**Keywords:** *Candida albicans*, *Galleria mellonella*, biofilm, muscarinic, pilocarpine hydrochloride, repurposing

## Abstract

Acetylcholine modulates the virulence of Candida albicans and regulates an appropriate immune response to infection in a Galleria mellonella infection model. Indeed, the evidence suggests that C. albicans possesses a functional cholinergic receptor that can regulate filamentous growth and biofilm formation. Furthermore, G. mellonella immune cell subsets possess repertories of cholinergic receptors which regulate an effective and appropriate cellular immune response to C. albicans infection. This study aimed to investigate the cholinergic receptor subtype involved in regulation of filamentous growth and biofilm formation by C. albicans and determine the roles of cholinergic receptors in modulation of G. mellonella immune cell subsets. The general muscarinic receptor agonist, pilocarpine hydrochloride, inhibited C. albicans biofilm formation and pathogenicity, a phenomenon that could be reversed using the general muscarinic receptor antagonist, scopolamine. Pilocarpine hydrochloride protected G. mellonella larvae from C. albicans infection via inhibition of C. albicans filamentation and appropriate regulation of cellular immunity. However, scopolamine abrogated the capacity of pilocarpine hydrochloride to protect G. mellonella larvae from C. albicans infection. Furthermore, acetylcholine and pilocarpine hydrochloride exhibited differential modulatory capabilities on Galleria mellonella hemocyte responses to C. albicans. The data in this article demonstrate that a muscarinic receptor modulates C. albicans filamentation and biofilm formation. Furthermore, the results suggest that G. mellonella hemocyte subsets possess unique repertoires of cholinergic receptors that regulate their differentiation, activation, and function in contrasting manners. Therefore, targeting cholinergic receptors by repurposing currently licensed cholinergic drugs may offer novel therapeutic solutions for the prevention or treatment of fungal infections.

**IMPORTANCE** Candida albicans is the most common human fungal pathogen with an estimated crude mortality rate of 40%. The ability of the organism to switch from the yeast to hyphal form and produce biofilms are important virulence factors. C. albicans infections are combatted by the host immune system. However, *Candida* triggers a strong inflammatory response that, if not appropriately regulated, can damage host tissues. Therefore, it is important that the host immune response eliminates the fungus but limits tissue damage. This study provides evidence that targeting cholinergic receptors cannot only curb the virulence of C. albicans by inhibiting filamentous growth and biofilm formation but can also appropriately regulate the host immune response to induce rapid clearance with limited damage to vital tissues. This article provides evidence that repurposing licensed drugs that target cholinergic receptors may offer novel therapeutic solutions for the prevention or treatment of fungal infections.

## Abstract (precis)

Candida albicans is the most common human fungal pathogen with an estimated crude mortality rate of 40%. The ability of the organism to switch from the yeast to hyphal form and produce biofilms are important virulence factors. C. albicans infections are combatted by the host immune system. However, *Candida* triggers a strong inflammatory response that, if not appropriately regulated, can damage host tissues. Therefore, it is important that the host immune response eliminates the fungus but limits tissue damage. This study provides evidence that targeting cholinergic receptors cannot only curb the virulence of C. albicans by inhibiting filamentous growth and biofilm formation but can also appropriately regulate the host immune response to induce rapid clearance with limited damage to vital tissues. This article provides evidence that repurposing licensed drugs that target cholinergic receptors may offer novel therapeutic solutions for the prevention or treatment of fungal infections.

## INTRODUCTION

Acetylcholine (ACh) is synthesized by almost every cell of the human body, and its functions go way beyond those of a classical neurotransmitter (1–3). ACh is known to modulate pathogen-driven immune responses, downregulating potentially damaging chronic inflammation and promoting favorable disease outcomes in selected *in vivo* models of bacterial sepsis (4–7). Furthermore, human immune cells express both nicotinic and muscarinic acetylcholine receptors (nAChRs and mAChRs). These receptors have been demonstrated to modulate cellular immunity against bacterial pathogens via cholinergic-dependent mechanisms (2, 8–10).

Evidence suggests that bacteria and fungi are capable of synthesizing ACh (11–13). However, very little is known about the cholinergic receptor repertoire of these microorganisms. Several bacterial species possess homologs of mammalian nicotinic receptors (14, 15), although the functional roles of these receptors have yet to be elucidated. To date, there are no studies that have identified or characterized fungal cholinergic receptors. However, sequencing of the Candida albicans genome has suggested that this organism possesses putative cholinergic receptor genes (16).

Acetylcholine has been demonstrated to promote favorable disease outcomes to C. albicans infection in a Galleria mellonella infection model (17). Acetylcholine modulates the pathogenicity of C. albicans by inhibiting morphogenesis, biofilm formation, and the expression of virulence factors. In addition, ACh promotes an effective cellular immune response to fungal infection, facilitating rapid clearance from infected tissues and affording protection from chronic-inflammation-induced damage of vital tissues (17).

The innate immune system plays a crucial role in protection against systemic C. albicans infections, as evidenced by the fact that immunocompromised, critically ill, and elderly patients show increased susceptibility. Insects lack an acquired immune system but possess a complex and effective innate immune system, which can be divided into humoral and cellular defense components. The cellular defenses are defined as hemocyte-mediated responses and involve processes such as phagocytosis and encapsulation (18). G. mellonella possesses at least six hemocyte subtypes. However, plasmatocytes and granulocytes are the most abundant circulating cells in G. mellonella hemolymph (19). These cells have similar characteristics to human neutrophils (20) and the ability to respond to ACh (17). Human immune cells, including neutrophils, possess various repertoires of nAChRs and mAChRs, and activation of specific receptors can have different consequences for immune function (21–23).

Advancements in our understanding of human cholinergic receptors and their roles in various pathologies have led to the discovery of a plethora of small-molecule agonists and antagonists with therapeutic potential. Many of these molecules have been developed for treatment of conditions whose pathology is defined by loss or gain of cholinergic function, such as neurodegenerative disorders (24). However, a variety of cholinergic drugs have been utilized to research the effects of cholinergic receptors on nonneurological pathologies, including bacterial sepsis, and investigated for their therapeutic effectiveness in inflammatory disease (25–27).

Repositioning of cholinergic drugs for the treatment of candidiasis may provide new avenues for therapeutic strategies. Hence, the aims of this study were to begin to delineate the cholinergic receptor subtype responsible for the modulation of biofilm formation by C. albicans and to investigate in further detail the role of cholinergic receptor subtypes on cellular immunity against C. albicans infection in a G. mellonella infection model.

## RESULTS

### Pilocarpine hydrochloride specifically inhibits Candida albicans biofilm formation and pathogenicity through interaction with a muscarinic-like receptor.

Acetylcholine inhibits C. albicans biofilm formation (17). Therefore, the effect of a nonspecific nicotinic receptor agonist, SIB1508Y maleate (SIBm), and a nonspecific muscarinic receptor agonist, pilocarpine hydrochloride (PHCl), on C. albicans biofilm formation was investigated to determine whether this was due to activation of a specific subtype of cholinergic receptor.

**FIG 1:**
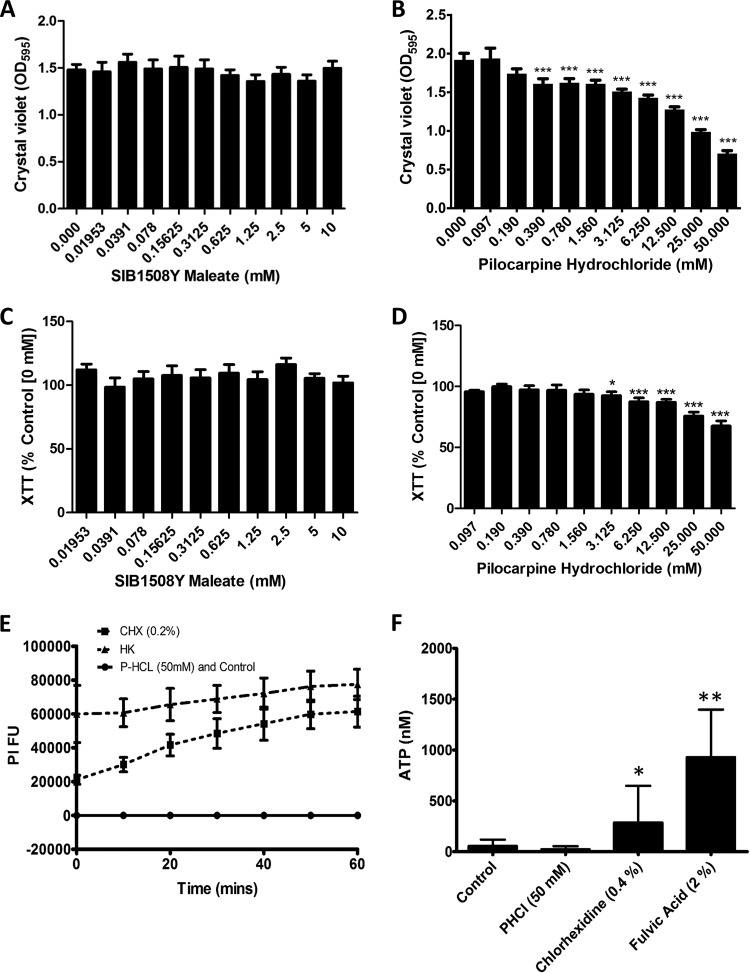
The general muscarinic receptor agonist pilocarpine hydrochloride inhibits Candida albicans biofilm formation *in vitro*. (A and B) Biofilm biomass was assessed using the crystal violet assay after C. albicans was cultured for 24 h in RPMI 1640 containing different concentrations of SIBm (0 to 10 mM) (A) and PHCl (0 to 50 mM) (B). Data are expressed as raw OD_595_ values, and the bars represent the mean values (plus standard deviations [SD] [error bars]) from triplicate wells of six independent experiments (*n* = 6). (C and D) Biofilm metabolic activity was assessed using the XTT assay after C. albicans was cultured for 24 h in RPMI 1640 containing different concentrations of SIBm (0 to 10 mM) (C) and PHCl (0 to 50 mM) (D). Data are expressed as percent metabolic activity compared to untreated controls (0 mM PHCl), and the bars represent the mean values (+SD) from triplicate wells of six independent experiments (*n* = 6). For panels A to D, values that are significantly different compared to the control values (0 mM SIBm or PHCl) are indicated by asterisks as follows: *, *P < *0.05; **, *P < *0.01; ***, *P < *0.001. (E and F) The effect of PHCl on the permeability of the C. albicans cell wall was investigated using a propidium iodide (PI) uptake (E) and ATP release assay (F). For the PI uptake assay, data are shown as fluorescence intensity units, and the bars represent the mean values (+SD) from triplicate wells of three independent experiments (*n* = 3). Heat-killed (HK) and chlorhexidine (CHX) (0.2%)-treated C. albicans were included as positive controls, and cells in RPMI 1640 alone were included as a negative control. For the ATP release assays, data are shown as nanomolar concentrations of ATP release, and the bars represent the mean values (+SD) from triplicate wells of three independent experiments (*n* = 3). Chlorhexidine (0.2%)- and fulvic acid (2.0%)-treated C. albicans cells were included as positive controls, and cells in RPMI 1640 alone were included as a negative control. *, *P < *0.05; **, *P < *0.01 compared to the control (cells in RPMI 1640 alone).

Biomass quantification assays revealed that SIBm had no effect on C. albicans biofilm formation *in vitro* (Fig. 1A). In contrast, PHCl caused a dose-dependent decrease in biofilm biomass, with statistically significant reductions observed with concentrations ranging between 0.39 and 50 mM (all *P < *0.001) (Fig. 1B). The XTT metabolic assay revealed that SIBm had no effect on C. albicans metabolic activity (Fig. 1C). However, a slight but significant reduction in C. albicans metabolic activity compared to the untreated control (0 mM) was observed when treated with PHCl concentrations ranging between 3.125 and 50 mM (all *P < *0.01) (Fig. 1D).

**FIG 2:**
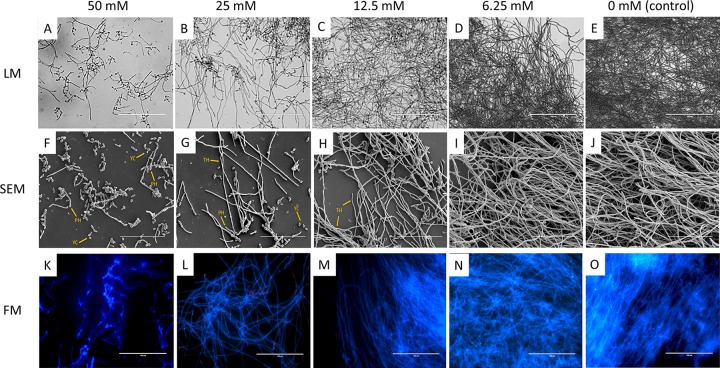
The general muscarinic receptor agonist pilocarpine hydrochloride inhibits filamentation and biofilm formation *in vitro* without affecting cell viability. (A to J) To assess the effects of PHCl on C. albicans morphology and viability, microscopy was employed. Light microscopy (LM) (A to E) and scanning electron microscopy (SEM) (F to J) was performed to visualize changes in biofilm biomass and C. albicans cell morphology after culture for 24 h in RPMI 1640 containing different concentrations of PHCl (0 to 50 mM). Representative images are shown from duplicate coverslips of three independent experiments. YC, yeast cells; PH, pseudohyphae; TH, true hyphae. Bars, 100 µm (A to E) and 700 µm (F to J). (K to O) Fluorescence microscopy (FM) was performed to assess cell viability. C. albicans was cultured for 24 h in RPMI 1640 containing different concentrations of PHCl, and viability was assessed using calcofluor white (blue) and propidium iodide (red) staining. Representative images are shown from duplicate coverslips of three independent experiments. Bars, 100 µm.

Reductions in metabolic activity are associated with reduced biofilm formation but can also be attributed to cell death. A planktonic MFC was performed according to the CLSI M-27A broth microdilution methodology (28) and revealed that none of concentrations of SIBm or PHCl investigated possessed fungicidal activity (data not shown). In addition, PHCl had no destabilizing effects on the C. albicans cell wall as observed by measuring both PI uptake (Fig. 1E) and ATP release (Fig. 1F). Microscopy was also employed to further ensure that PHCl was specifically inhibiting filamentation and not affecting cell viability. Light microscopy (LM) (Fig. 2A to E) revealed that PHCl inhibited filamentation and biofilm formation in a dose-dependent manner. Furthermore, visually, in the presence of increasing concentrations of PHCl, more C. albicans cells maintained a yeast morphology, suggesting that PHCl was inhibiting the yeast-to-hypha transition. Scanning electron microscopy (SEM) analysis (Fig. 2F to J) further confirmed the fact that PHCl inhibited biofilm formation due to inhibition of the yeast-to-hypha transition in a dose-dependent manner. Fluorescence microscopy (FM) (Fig. 2K to O) with CFW (blue) and PI staining (red) revealed no visible differences in cell viability between any of the concentrations of PHCl investigated and therefore confirmed that PHCl was not toxic to C. albicans at the concentrations used in this study.

**FIG 3:**
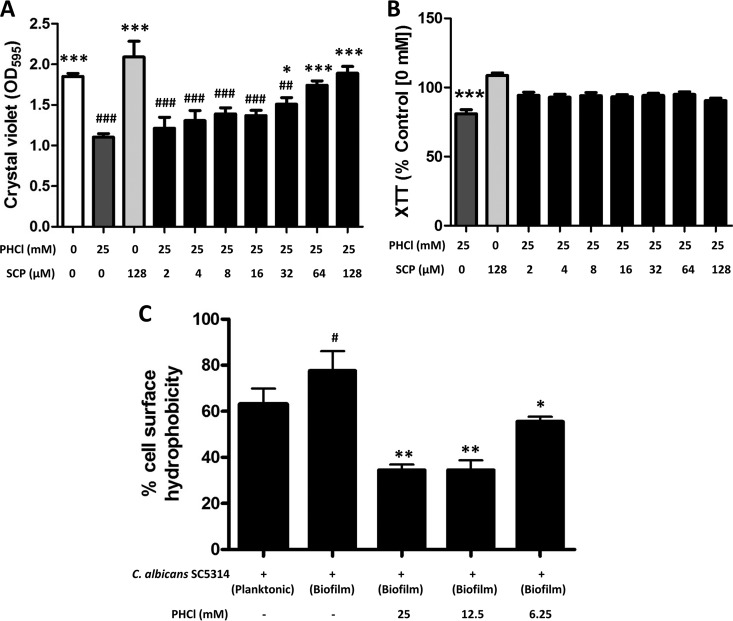
Pilocarpine hydrochloride acts through a specific muscarinic-like receptor to inhibit biofilm formation and modulate cell wall hydrophobicity. (A) Biofilm biomass was assessed using the crystal violet assay after C. albicans was cultured for 24 h in RPMI 1640 containing 25 mM PHCl and different concentrations of the nonspecific muscarinic receptor antagonist scopolamine (SCP) (0 to 128 µM). Data are expressed as raw OD_595_ values, and the bars represent the mean values (+SD) from triplicate wells of three independent experiments (*n* = 3). Candida albicans was cultured in the absence of any compound, in the presence of PHCl alone, and in the presence of SCP alone as controls. #, significantly different from cells cultured in the absence of any compound; *, significantly different from cells cultured in PHCl alone; * or #, *P* < 0.05; ** or ##, = *P* < 0.01; *** or ###, *P* < 0.001. (B) Biofilm metabolic activity was assessed using the XTT assay after C. albicans was cultured for 24 h in RPMI 1640 containing 25 mM PHCl and different concentrations of the nonspecific muscarinic receptor antagonist scopolamine (SCP) (0 to 128 µM). Data are expressed as percent metabolic activity compared to untreated controls (0 mM PHCl or SCP), and the bars represent the mean values (+SD) from triplicate wells of three independent experiments (*n* = 3). Candida albicans cultured in the presence of PHCl and SCP alone acted as controls. *, significantly different from cells cultured in SCP alone; ***, *P* < 0.001. (C) Cell wall hydrophobicity was assessed using the MATH assay (29). The bars represent the mean values (+SD) from duplicate samples of five independent experiments (*n* = 5). #, significantly different from cells cultured planktonically; *, significantly different from cells cultured as a biofilm in the absence of PHCl.

To ensure that PHCl was specifically targeting a receptor with homology to human muscarinic receptors, biofilm biomass and metabolic activity assays were repeated in the presence of different concentrations of the nonspecific muscarinic receptor antagonist scopolamine (SCP). SCP inhibits the PHCl-induced reduction in biofilm formation in a dose-dependent manner with concentrations ranging from 64 to 128 µm, revealing no significant differences in biofilm biomass in the presence of 25 mM PHCl compared to the control (Fig. 3A). Furthermore, all concentrations of SCP abolished any PHCl-induced reductions in metabolic activity (Fig. 3B).

As hydrophobicity is a characteristic related to C. albicans biofilm formation (29), the effect of PHCl on C. albicans cell surface hydrophobicity was investigated using the microbial adhesion to hydrocarbon (MATH) assay. A statistically significant increase in cell surface hydrophobicity of C. albicans cells cultured as a biofilm compared to planktonic cells was observed (*P < *0.05) (Fig. 3C). Interestingly, when C. albicans biofilms were cultured in the presence of 25, 12.5 (both *P < *0.01), and 6.25 (*P < *0.05) mM PHCl, there was a statistically significant decrease in cell surface hydrophobicity (Fig. 3C).

### Pilocarpine hydrochloride specifically modulates the pathogenesis of Candida albicans infection in a Galleria mellonella model.

**FIG 4:**
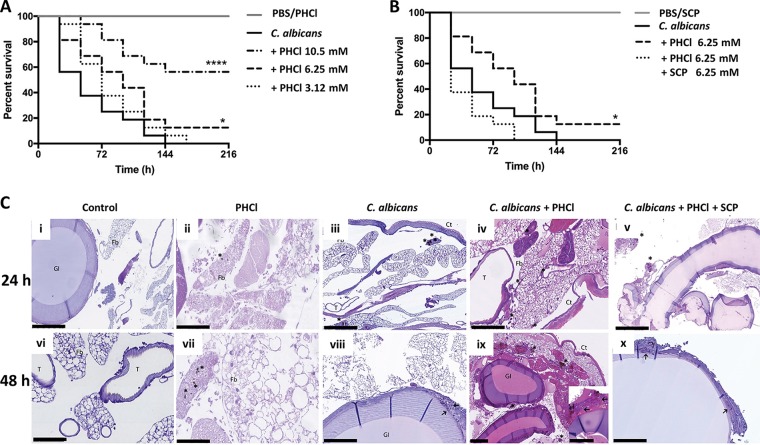
Pilocarpine hydrochloride acts through muscarinic-like receptors to prolong survival of Candida albicans-infected Galleria mellonella by inhibiting biofilm formation and modulating host immunity *in vivo*. The effect of PHCl on the outcome of systemic candidiasis was investigated using a G. mellonella infection model. (A) A Kaplan-Meier plot shows the effects of different concentrations of PHCl on the survival of Candida albicans-infected larvae. The data are derived from three independent experiments with groups of 16 larvae (*n* = 48). ***, P < 0.001; *, P < 0.05, as determined by the log rank test in comparison to larvae inoculated with C. albicans alone. PBS alone and PHCl alone-injected larvae were used as controls and had no effect on larvae survival. (B) To verify specificity, C. albicans-infected larvae were also inoculated with PHCl and SCP in combination. The data are derived from three independent experiments with groups of 16 larvae (*n* = 48). *, *P < *0.05, as determined by the log rank test in comparison to larvae inoculated with C. albicans. Larvae injected with PBS alone and SCP alone were used as controls, and PBS and SCP alone had no effect on larva survival. (C) Histological analysis of larvae was performed at 24 and 48 h postinoculation using hematoxylin and eosin (HE) and periodic acid-Schiff (PAS) staining. (i and vi; HE) (ii and vii; HE) larva inoculated with PHCl (6.25 mM) alone. (iii and viii; PAS) larvae infected with C. albicans. (iv and ix; HE) larvae infected with C. albicans in the presence of PHCl (6.25 mM). (v and x; PAS). Larvae infected with C. albicans in the presence of PHCl (6.25 mM) and SCP (6.25 mM). Asterisks highlight melanized nodules, whereas arrows show C. albicans cells and hyphae. Representative images are shown from histological analysis of two larvae for each condition from three independent experiments. Fb, fat body; Ct, cuticle; GI, gastrointestinal tract; T, trachea; Nd, nodule. Bars, 250 μm (panels i, v, and ix) and 100 μm (panels ii, iii, iv, vi, vii, viii, and x).

Biofilm formation is associated with C. albicans pathogenicity, and ACh has previously been shown to protect G. mellonella larvae from C. albicans*-*induced mortality (17). Therefore, the effect of PHCl on C. albicans pathogenicity *in vivo* was investigated using a G. mellonella killing assay. PHCl alone (10.5 mM) had no adverse effects on survival of the larvae (Fig. 4A). Indeed, PHCl protects G. mellonella larvae from C. albicans*-*induced mortality in a dose-dependent manner compared to larvae inoculated with C. albicans alone observed using 10.5 and 6.25 mM PHCl (*P < *0.001 and *P < *0.05, respectively; as determined using the log rank test) (Fig. 4A). Furthermore, SCP (6.25 mM) alone had no adverse effects on the survival of the larvae. In fact, in the presence of PHCl and SCP, the survival of the larvae was comparable to those inoculated with C. albicans alone, suggesting that SCP inhibited the PHCl-induced protection against C. albicans infection (Fig. 4B).

To visualize the effects of PHCl alone and in combination with SCP on C. albicans pathogenicity and G. mellonella hemocyte responses to infection, *in vivo* histological analysis was performed. Control sham-injected larvae after both 24 and 48 h demonstrated the presence of small hemocyte aggregates, adjacent to the gut and the tracheal system, resembling human MALT (mucosa-associated lymphoid tissue) and BALT (bronchus-associated lymphoid tissue). Furthermore, some hemocytes were also detected close to the fat body, near hemocoel cavities and distributed as a monolayer in the subcuticular areas (Fig. 4C, panels i and vi).

Twenty-four hours postinoculation, the hemocyte response in PHCl-injected larvae was characterized by uniformly dispersed small melanized nodules and an increase in circulating hemocytes (Fig. 4Cii). SCP alone resulted in poor immune activation and failed to induce melanization (data not shown). In larvae inoculated with C. albicans alone, the hemocyte response was characterized by melanized nodules of intermediate size, mainly located in the subcuticular area and in the fat body, with nodules found only rarely in paratracheal areas (Fig. 4Ciii). In contrast, the hemocyte response in larvae inoculated with C. albicans plus PHCl was characterized by the presence of single melanized hemocytes or very small aggregates of hemocytes with melanin deposition surrounding yeast cells. These aggregates were uniformly distributed in the hemolymph, close to the fat body and in peritracheal tissues. In addition, no filamentous growth was detected (Fig. 4Civ). In contrast, larvae inoculated with C. albicans SC5314 and treated with PHCl and SCP showed the presence of medium-sized nodules with scanty melanization and poor hemocyte recruitment into invaded tissues. Furthermore, poorly melanized yeast cells and hyphae were seldom detected in the nodules (Fig. 4Cv), similar to larvae infected with C. albicans alone (Fig. 4Ciii).

Forty-eight hours postinoculation, PHCl-injected larvae showed very few and small nodules with faint melanization (Fig. 4Cvii). Larvae infected with C. albicans alone showed increased hemocytes in the subcuticular, intestinal, and paratracheal areas with large nodules and multifocal melanization and heavy damage of the fat body. Hyphal invasion of the intestinal walls, and to a lesser extent, of the bronchial system was also detected (Fig. 4Cviii). In contrast, larvae infected with C. albicans plus PHCl exhibited decreased inflammation and less aggressive fungal infiltration of vital larval tissues, with only small melanized nodules mainly distributed in subcuticular areas. In addition, C. albicans hypha formation was not observed, and there was less microvacuolization of the fat body (Fig. 4Cix). When treated with both PHCl and SCP, C. albicans infection resulted in hyphal invasion of the intestinal walls (Fig. 4Cx), with a histological picture similar to larvae infected with C. albicans alone (Fig. 4Cviii).

### Pilocarpine hydrochloride and acetylcholine differentially modulate hemocyte responses to C. albicans both *in vitro* and *in vivo*.

**FIG 5:**
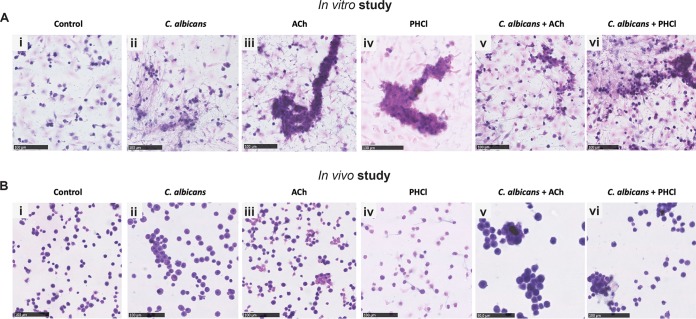
Effects of pilocarpine hydrochloride and acetylcholine on hemocyte responses to C. albicans *in vitro* and *in vivo*. (A) In the *in vitro* study, hemocytes isolated from untreated larvae were either left unstimulated (control) (i) or stimulated with C. albicans (ii), ACh (iii), PHCl (iv), C. albicans plus ACh (v), and C. albicans plus PHCl for 24 h. (B) In the *in vivo* study, larvae were bled 24 h after sham inoculation with PBS (control) (i) or inoculation with C. albicans (ii), ACh (iii), PHCl (iv), C. albicans plus ACh (v), and C. albicans plus PHCl. Representative images are shown from hematoxylin and eosin staining of hemocytes from three larvae for each condition from three independent experiments. Bars, 100 μm.

It has previously been demonstrated that ACh promotes G. mellonella hemocyte function (17). Therefore, the effects of PHCl on hemocyte cellularity, subtypes, and nodule formation during the pathogenesis of C. albicans infection were assessed and compared to the effects of ACh (Fig. 5 and Table 1).

**TABLE 1:** Effects of pilocarpine hydrochloride and acetylcholine on hemocyte cellularity and subtype characteristics *in vitro* and *in vivo*

Condition and treatment^*a*^	Cellularity	Hemocyte subset^*b*^							Nodule		*C. albicans*^*c*^
		Pr	Gr	Pl	Co	Sp	Ad	Oe	Size	Melanization	
*In vitro*											
CTR	Low	+	+	−	−	+	+	−	−	−	−
SC5314	High	+	++	++	++	++	+	+	Medium	++	+++
ACh	High	+	++	+++	+	−	−	−	Medium	−	−
PHCl	Low	+	+	−	−	+	+	−	−	−	−
SC5314/ACh	High	+	+++	+++	++	+	+	−	Large	−	+
SC5314/PHCl	High	+	+	+++	+	+	++	+	Small	−	+

*In vivo*											
CTR	Low	+	+	−	−	+	+	−	−	−	−
SC5314	High	+	+++	++	++	++	+	+	Large	+++	+++
ACh	Intermediate	+	++	+++	+	−	−	−	Medium	−	−
PHCl	Low	+	+	+	−	+	+	−	−	−	−
SC5314/ACh	Intermediate	+	+++	+++	++	+	+	+	Large	−	+
SC5314/PHCl	Intermediate	+	++	+++	++	+	+	+	Small	−	+

a *Galleria mellonella* larvae were inoculated with PBS (control [CTR]), C. albicans SC5314, acetylcholine (ACh), pilocarpine hydrochloride (PHCl), C. albicans plus ACh (SC3514/ACh), and C. albicans plus PHCl (SC5314/PHCl).

b Pr, prohemocytes; Gr, granulocytes; Pl, plasmatocytes; Co, coagulocytes; Sp, spherulocytes; Ad, adipocytes; Oe, oenocytes. Immune cell subtype quantification was scored as follows: −, absent/rare; +, 1 to 10%; ++, 11 to 30%; +++, 31 to 50%.

c C. albicans presence was quantified as follows: −, absent; +, few cells; ++, multiple yeast agglomerate usually embedded in nodules; +++, abundant yeasts and/or hyphae with widespread diffusion in nodules.

An increase in hemocyte cellularity with a predominance of plasmatocytes, granulocytes, and spherulocytes was observed 24 h after exposure to C. albicans alone *in vitro*. The hemocytes formed small, mainly two-dimensional, melanized nodules. Furthermore, aggregates of yeast cells and hyphae were observed, as well as coagulation fibers similar to neutrophil extracellular traps (NETs) (Fig. 5Aii and Table 1). ACh alone induced an increase in cellularity, with a predominance of plasmatocytes and granulocytes, but no spherulocytes. Cell aggregation was more evident as well as multidimensional nodule formation with no visible melanization (Fig. 5Aiii and Table 1). Similarly, PHCl induced hemocyte aggregation, leading to multidimensional nodule formation. However, nodules were smaller and consisted mainly of plasmatocytes, a few granulocytes, and no spherulocytes (Fig. 5Aiv and Table 1). In the presence of C. albicans plus ACh, there was an increase in cellularity with a predominance of plasmatocytes, granulocytes, and spherulocytes. A strong induction of aggregation and multidimensional nodule formation with no melanization was also clearly visible (Fig. 5Av and Table 1). In contrast, although an increase in cellularity was also evident in C. albicans-plus-PHCl treated hemocytes, granulocyte and spherulocyte numbers were reduced compared with cells treated with C. albicans plus ACh. There was also limited cell aggregation and the formation of small two-dimensional nodules with no melanization (Fig. 5Avi and Table 1).

To determine whether the effects observed *in vitro* could be differently affected *in vivo* due to tissue secretion of regulatory molecules, the same analysis was performed on hemocytes isolated from G. mellonella larvae 24 h after infection with C. albicans in the presence and absence of ACh or PHCl. Sham-infected larvae were used as a control (Fig. 5Bi and Table 1). Comparison with the control cells *in vitro* (Fig. 5Ai and Table 1) revealed a greater number of cells with finely vacuolated cytoplasm, resembling spherulocytes and adipocytes. For all experimental conditions, the *in vivo* observations substantially overlapped the *in vitro* observations. However, overall cellularity was slightly reduced *in vivo* compared to *in vitro* due to tissue sequestration of hemocytes. With respect to specific treatments, hemocytes from C. albicans-infected larvae revealed a predominance of granulocytes and spherulocytes (Fig. 5Bii and Table 1) compared to hemocytes stimulated with C. albicans alone *in vitro*. In addition, the granulocytes were larger and contained large vacuoles within melanized cytoplasm compared with the small two-dimensional melanized nodules observed in the cells *in vitro* (Fig. 5Aii and Table 1). Hemocytes from larvae inoculated with ACh or PHCl alone and with C. albicans plus PHCl (Fig. 5Biii, iv, and vi, respectively; Table 1) showed similar cellularity and differentiation properties to hemocytes exposed to the same compound *in vitro* (Fig. 5Aiii, iv, and vi, respectively; Table 1). In contrast, hemocytes isolated from larvae infected with C. albicans plus ACh (Fig. 5Bv and Table 1) showed increased melanization compared to hemocytes cultured *in vitro* and infected with C. albicans alone (Fig. 5Av and Table 1).

## DISCUSSION

Acetylcholine has previously been found to inhibit C. albicans virulence, both *in vitro* and *in vivo*, leading to the hypothesis that C. albicans possesses putative cholinergic receptors (17, 30). ACh is a general cholinergic receptor agonist with activity against both nicotinic ACh receptors (nAChR) and muscarinic ACh receptors (mAChR). In this study, the general nicotinic agonist SIB1508Y maleate had no effect on C. albicans biofilm formation *in vitro*. In contrast, PHCl, a nonspecific muscarinic agonist, inhibited filamentation and biofilm formation. PHCl had no fungicidal activity, and the pharmacological specificity of the response was confirmed both *in vitro* and *in vivo* using a general muscarinic receptor antagonist, scopolamine. Therefore, C. albicans possesses an uncharacterized cholinergic receptor involved in regulating filamentation similar in phenotype to human muscarinic receptors.

PHCl is used clinically to treat xerostomia and glaucoma (31, 32). Pharmacological studies using *in vitro* and *in vivo* models indicate that, despite being a general muscarinic agonist, PHCl has a predominance for M3 and M1 (M3<M1) muscarinic receptors in mammals (33). Muscarinic receptors are G protein-coupled receptors specialized in responding to ligands involved in cell-cell communication (34). Fungi express a number of G protein-coupled receptors that play vital roles in sensing extracellular signals (34). In C. albicans, G protein-coupled receptors have been shown to regulate filamentation. However, current evidence suggests that G protein-coupled receptors promote filamentation and biofilm formation (34, 35). In addition, the general muscarinic receptor antagonist dicyclomine attenuates C. albicans hypha formation by upregulating *tup1* expression, the master negative regulator of hypha formation (36). However, in this study, dicycloamine was found to be toxic to C. albicans, and the authors did not discriminate between direct fungicidal activity and specific on target effects of the compound (36).

The data herein suggest that C. albicans possesses a G protein-coupled receptor that can negatively regulate filamentation and biofilm formation. To date, our knowledge of fungal G protein receptors and their roles in regulation of cellular phenotype is not complete, and a number of orphan receptors have still to be functionally characterized (34). However, human studies have shown that different muscarinic receptor subtypes can have opposing functions (37, 38). It is therefore feasible that C. albicans possesses more than one G protein-coupled receptor that can differentially modulate filamentation, biofilm formation, and virulence. Further research is required to confirm this hypothesis.

Both M1 and M3 receptors utilize intracellular calcium as a second messenger (39). There is evidence that suggests that unrestricted calcium uptake can inhibit C. albicans mycelial growth, which indicates a critical role for calcium in the regulation of C. albicans morphogenesis (40). Calcineurin is a major player in eukaryotic calcium-dependent signal transduction pathways. In C. albicans, the calcineurin pathway has been shown to be involved in tolerance to antifungal agents, cation homeostasis, and virulence. However, studies of the role of the calcineurin/calmodulin pathway in C. albicans hyphal growth and biofilm formation have revealed contradictory results (41). Calcineurin is a calcium-dependent serine/threonine-specific protein phosphatase, and studies have identified at least 15 novel downstream signaling targets in yeast (42). These downstream targets have been found to play roles in modulating the cell cycle, membrane structure, and cell wall integrity (42). In this study, PHCl was found to decrease C. albicans cell surface hydrophobicity. Cell surface hydrophobicity has been shown to be related to cell wall composition and is also a predictor of biofilm-forming capability in *Candida* species (43). Cell adhesion to host surfaces is regarded as a major virulence factor for C. albicans (44). Therefore, these findings suggest that cholinergic signaling inhibits C. albicans virulence by additional mechanisms, possibly regulated by intracellular calcium via the calcineurin/calmodulin pathway. However, further research is required to confirm this hypothesis.

C. albicans-triggered diseases represent an intriguing immunological paradigm as they result from a disrupted balance between tolerance and resistance by the immune system (45). The host immune response must eliminate the fungus while limiting collateral damage to tissues and restoring a homeostatic environment. The invasive growth of C. albicans, however, triggers a strong host inflammatory response that can damage infected organs (46). Acetylcholine has been found to promote rapid clearance of C. albicans in a G. mellonella infection model, while at the same time protecting against inflammation-induced tissue damage (17). The data in this article show that PHCl can also modulate host immunity to C. albicans infection with similar outcomes. Indeed, PHCl not only inhibited filamentation *in vivo* but could also promote rapid and effective clearance of the pathogen while limiting bystander vital tissue damage. Therefore, PHCl can also modulate the pathogenesis of C. albicans infection via muscarinic receptors on G. mellonella immune cell subsets.

Despite similarities between ACh and PHCl in terms of pathogenesis of a C. albicans infection in G. mellonella, distinct differences in the subtypes of hemocytes involved were noted. G. mellonella possesses at least six immune cell subsets, granulocytes, plasmatocytes, oenocytoids, spherulocytes, prohemocytes, and adipohemocytes (47). Granulocytes, the very first cells recruited to counteract pathogen invasion, were strongly activated *in vivo* in the presence of ACh, but not PHCl. Similarly, spherulocytes, whose function is still to be defined, were specifically recruited into tissues in ACh-inoculated larvae, but not in PHCl-inoculated larvae. As spherulocyte recruitment is known to be dependent on granulocyte recruitment, this finding is perhaps unsurprising. However, a direct inhibitory effect of PHCl on spherulocyte recruitment cannot be ruled out. In addition to hemocyte recruitment, PHCl was found to promote less hemocyte aggregation *in vivo* than ACh, and the resulting nodules were mainly characterized by the presence of plasmatocytes, which are usually necessary during the later phases of nodulation and encapsulation.

In this study, differences between *in vitro-* and *in vivo*-stimulated hemocytes, both in terms of morphology and quantity, were observed. In particular, under all conditions investigated, the *in vitro* spherulocyte content was reduced compared with the *in vivo* spherulocyte content, as spherulocytes do not originate from division of circulating hemocytes but are derived from the granulocyte lineage of hematopoietic organs (48). We hypothesize therefore that the biological functions of ACh and PHCl may also be modulated by larval tissue or plasma factors that are missing in cultured cells. Indeed, the fat body of G. mellonella plays a crucial metabolic function, producing antimicrobial peptides and proteins that are involved in dictating immune responses.

The data in this article suggest that cholinergic receptors can modulate G. mellonella hematopoiesis and differentiation. Interestingly, in humans, cholinergic receptors have been shown to regulate hematopoiesis (49). Platelet and megakaryocyte precursors (50), as well as myeloid and erythroid progenitors in the bone marrow (51), all express the α7nAChR which is suggested to play a vital role in regulating their differentiation and maturation. Therefore, it is interesting to speculate that similar systems operate in G. mellonella. However, further research using specific cholinergic receptor agonist and antagonists is required to begin to delineate the type of cholinergic receptors that regulate G. mellonella cellular immunity. The fact that ACh, and not PHCl, induced hemocyte recruitment and aggregation suggests that nicotinic receptors rather than muscarinic receptors may play a more important role in immune regulation in this model host.

In conclusion, the present study suggests that different cholinergic receptors may be involved in the promotion of favorable outcome to C. albicans systemic infection. A muscarinic-type receptor seems to modulate C. albicans filamentation and biofilm formation. In addition, the data suggest that hemocyte subsets of G. mellonella possess different repertoires of cholinergic receptors that can modulate their differentiation and function. Therefore, this article provides evidence that targeting cholinergic receptors by repurposing currently licensed cholinergic drugs may be a direct or adjunctive therapeutic strategy to prevent or treat potentially fatal fungal infections.

## MATERIALS AND METHODS

### Candida albicans yeast culture and biofilm formation.

C. albicans SC5314 was subcultured and propagated as described previously (17). C. albicans SC5314 was then standardized to 1 × 10^6^ cells/ml in Roswell Park Memorial Institute 1640 medium (RPMI 1640) in the presence or absence of various concentrations of pilocarpine hydrochloride (PHCl) (Tocris, UK) or SIB1508Y maleate (Tocris, UK). To determine specificity of action, experiments were also performed with PHCl in the presence or absence of various concentrations of scolopamine (SCP) (Tocris, UK). Standardized cells were allowed to form biofilms in flat-bottomed 96-well microtiter plates at 37°C for 24 h. Following incubation, metabolic activity was assessed using the 2,3-bis-(2-methoxy-4-nitro-5-sulfophenyl)-2*H*-tetrazolium-5-carboxanilide) (XTT) assay and biofilm biomass determined using the crystal violet assay as described previously (52). Experimental conditions were run in triplicate. Results are presented as mean values from at least four independent experiments.

### Propidium iodide uptake and ATP release assays.

To evaluate whether any indirect antifungal activity through the disruption of the cell membrane was induced by PHCl, we used a propidium iodide (PI) uptake and ATP release assay, as previously described (29). Briefly, C. albicans SC5314 was standardized to 5 × 10^7^ cells/ml in RPMI 1640 and treated with 50 mM PHCl for 60 min. After treatment, the supernatant was harvested, and the cells were washed with PBS and treated with 2 µM PI (in PBS). After incubation at 37˚C for 15 min, fluorescence was measured at excitation and emission wavelengths of 485 and 620 nm, respectively. The ATP release assay was performed according to the manufacturer’s instructions on supernatants harvested after 60 min using an ATP bioluminescent assay kit (Sigma-Aldrich, UK). Experimental conditions were run in triplicate and repeated on three independent occasions.

### Biofilm viability and cell morphology.

Standardized C. albicans SC5314 (1 × 10^6^ cells/ml) were inoculated in RPMI with or without PHCl on Thermanox coverslips (13 mm) within a 24-well tissue culture plate and then incubated for 24 h at 37°C. For light microscopy, coverslips were washed gently with PBS and stained with crystal violet (0.05% [vol/vol]). For fluorescence microscopy, the coverslips were washed gently with PBS and stained according to the manufacturers’ instructions with 5 μM calcofluor white (CFW) (Invitrogen, UK) and 20 μM propidium iodide (PI) (Sigma, UK). For scanning electron microscopy (SEM), the specimens were prepared as previously described (53). Biofilms were visualized under a fluorescence microscope (Motic BA400 Colorview system) in normal light mode for crystal violet-stained coverslips or at Ex_350_/Em_400_ for calcofluor white-stained coverslips and Ex_540_/Em_525_ for propidium iodide-stained coverslips. For SEM, biofilms were visualized by using a JEOL JSM-6400 scanning electron microscope. Representative images from 10 fields were taken. Experiments were repeated in duplicate on at least three independent occasions, and representative images are shown.

### Microbial adhesion to hydrocarbon assay.

The ability of yeast cells to adhere to a hydrocarbon source (Octane; Sigma, Italy) was used to measure cell surface hydrophobicity (CSH), as previously described (43). Briefly, C. albicans SC5314 was cultured in YPD (planktonic cells), RPMI 1640 alone (biofilm), or RPMI 1640 with 6.25, 12.5, and 25 mM PHCl for 24 h. The cells were then standardized to 10^8^ cells/ml and mixed with octane for 2 min. The aqueous and organic phases were allowed to separate for 10 min at 30°C, and the optical density of the aqueous phase was determined at OD_600_. The CSH index was calculated as follows: percent CSH = [1 − (*B* − *A*)] × 100 (where *A* is the initial absorbance of the aqueous phase, and *B* is the absorbance of the aqueous phase after partitioning). Samples were run in duplicate. Results are presented as mean values from five independent experiments.

### Galleria mellonella killing assay.

Pathogenicity of C. albicans SC5314 with or without PHCl was assessed using the G. mellonella killing assay as described previously (17). Sixteen randomly selected sixth-instar G. mellonella larvae (Allevamento Cirà, Como, Italy) with a body weight of between 200 to 300 mg were employed for each experimental group. Overnight YPD cultures of C. albicans SC5314 were standardized in PBS to the desired cell density. Larvae were inoculated into the hemocoel with 5 × 10^5^ cells/larva with or without PHCl at different concentrations using a 10-μl Hamilton syringe with a 26-gauge needle. In addition, larvae inoculated with PBS and PHCl alone were included for control purposes. A group injected simultaneously with PHCl with or without SCP was further added to verify the specificity of PHCl action. The inoculated larvae were incubated at 37°C, and the number of dead larvae was scored daily. All experiments were repeated on three independent occasions.

### Hemocyte characteristics.

For the *in vitro* experiments, the hemolymph of five G. mellonella larvae was collected into ice-cold Grace’s medium (Sigma-Aldrich, Italy) by lateral bleeding corresponding to the last right proleg. Hemocytes were counted and plated onto coverslips in a 24-well plate. The plates were incubated for 2 h to allow hemocytes to adhere prior to stimulation with C. albicans SC5314 (10^2^ yeast cells/well) for 24 h in the presence and absence of PHCl or ACh. After treatment, hemocytes were fixed in 4% paraformaldehyde and stained with hematoxylin and eosin. Hemocytes incubated in media alone acted as a control.

For the *in vivo* experiments, G. mellonella larvae were inoculated using the same conditions described for the killing assay. Twenty-four hours after treatment, hemocytes were isolated from the larvae as described above. Hemocytes from three larvae for each experimental condition were then pooled and plated onto coverslips in a 24-well plate. The coverslips were centrifuged for 3 min at 300 × *g* at room temperature and fixed in 4% paraformaldehyde and stained with hematoxylin and eosin.

In both *in vitro* and *in vivo* experiments, hemocyte proliferation (cellularity), subtype differentiation and nodule formation were assessed in comparison to hemocytes incubated in media only (*in vitro*) or hemocytes from sham-inoculated larvae (*in vivo*) A single trained and experienced pathologist (M. Falleni) carried out all characterization and quantification.

### Larval histology.

After hemolymph extraction, the same larvae were processed for histology as previously described (54). Briefly, the larvae were inoculated with buffered formalin and processed by means of transverse cut serial sections. Tissue sections were embedded in paraffin and routinely processed for conventional histopathology. Serial 4-µm tissue sections were stained with hematoxylin and eosin (HE) or periodic acid-Schiff stain (PAS). Image acquisition was performed by the NanoZoomer-XR C12000 series (Hamamatsu Photonics). To investigate C. albicans filamentation and the effect on cellular immunity *in vivo*, two time points were used, 24 and 48 h. Data were confirmed in three independent experiments, and representative images are shown.

### Statistical analysis.

Graph production, data distribution, and statistical analysis were performed using GraphPad Prism (version 4; La Jolla, CA). Crystal violet and XTT data were found to be abnormally distributed; therefore, all concentrations were compared to the control using a Kruskal-Wallis nonparametric test with a Dunns post-test. The ATP assay data were found to be normally distributed; therefore, a Tukey’s multiple-comparison test was used for statistical analysis. The G. mellonella survival curves were analyzed using a log rank test. Statistical significance was achieved if *P < *0.05.
